# Effect of standardized post-coercion review on subjective coercion: Results of a randomized-controlled trial

**DOI:** 10.1192/j.eurpsy.2021.2256

**Published:** 2021-12-07

**Authors:** A. Wullschleger, A. Vandamme, J. Mielau, L. Stoll, A. Heinz, F. Bermpohl, A. Bechdolf, M. Stelzig, O. Hardt, I. Hauth, V. Holthoff-Detto, L. Mahler, C. Montag

**Affiliations:** 1 Department of Psychiatry and Psychotherapy, Berlin Institute of Health, Charité – Universitätsmedizin Berlin, Freie Universität Berlin, Humboldt-Universität zu Berlin, Berlin, Germany; 2 Division of Adult Psychiatry, Department of Psychiatry, Geneva University Hospitals, Thônex, Switzerland; 3 Department of Psychiatry, Psychotherapy and Psychosomatics, Vivantes Hospital Am Urban and Vivantes Hospital im Friedrichshain/Charité Medicine Berlin, Berlin, Germany; 4 Department of Psychiatry and Psychotherapy, University of Cologne, Cologne, Germany; 5 Department of Psychiatry, Psychotherapy and Psychosomatics, Vivantes Wenckebach Clinic, Berlin, Germany; 6 Department of Psychiatry, Psychotherapy and Psychosomatics, Vivantes Clinic Neukölln, Berlin, Germany; 7 Department of Psychiatry, Psychotherapy and Psychosomatics, St. Joseph Hospital Berlin-Weissensee, Berlin, Germany; 8 Department of Psychiatry, Psychotherapy and Psychosomatics, Alexianer Hospital Hedwigshöhe, St. Hedwig Kliniken Berlin, Berlin, Germany; 9 Medical Faculty, University of Technology, Dresden, Germany; 10 Department of Psychiatry, Clinics in the Theodor-Wenzel-Werk, Berlin, Germany

**Keywords:** Coercion, post-coercion review, psychiatry, subjective coercion

## Abstract

**Background:**

Post-coercion review has been increasingly regarded as a useful intervention in psychiatric inpatient setting. However, little is known about its effect on perceived coercion.

**Methods:**

A multicenter, two-armed, randomized controlled trial was conducted, aiming at analyzing the effect of post-coercion review on perceived coercion. People with severe mental disorders, who experienced at least one coercive measure during inpatient treatment, were randomized using Zelen’s design to an intervention group receiving standardized post-coercion review, or a control group treated as usual. The MacArthur admission experience scale (AES) and the coercion ladder (CL) were used to assess perceived coercion during inpatient treatment. The coercion experience scale (CES) measured experienced coercion during the coercive intervention. Analyses of covariance were performed to determine group differences.

**Results:**

Of 422 randomized participants, *n* = 109 consented to participate in the trial. A restricted intention-to-treat analysis of all individuals who consented revealed no significant effect of the intervention on perceived coercion. A significant interaction effect between the factors gender and intervention on the AES scores was found. Sensitivity analysis revealed significant effects of the intervention on both AES and CL scores and an interaction effect between intervention and gender, indicating a higher efficacy in women. No effect of the intervention on CES scores was found.

**Conclusions:**

Standardized post-coercion review sessions did not alleviate the subjective perception of coercion in the total sample. However, post hoc analysis revealed a significant effect of the intervention in women. Results indicate the need to further address gender-specific issues related to coercion.

## Introduction

The use of coercive interventions such as seclusion and mechanical restraint in psychiatric settings and their consequences have been intensively debated during the last decades, especially since the adoption of the UN Convention on the Rights of People with Disabilities came into force [1]. In this context, subjectively perceived coercion has been investigated as important outcome. Associated with poor clinical outcomes, a negative impact on outpatient treatment [2,3] as well as with low satisfaction and negative attitudes toward hospital treatment [4]. Perception of fairness during the treatment process and participation in decision-making seem to mitigate the subjective perception of coercion [5–8]. Previous works suggested that women might be more prone to experience higher levels of perceived coercion than men [9,10], and that younger patients might experience higher levels of subjective coercion than older patients [11,12].

Among interventions aiming to reduce the use of coercive measures, post-coercion review has received growing attention. Post-coercion review sessions have been integrated into guidelines addressing the management of coercion [13]. However, such interventions are to date not sufficiently implemented [14]. Moreover, a clear definition of a post-coercion review or standards regarding their setting and content do not exist [14,15]. Interventions targeting both service users and staff members are needed to ensure a reflexive process and the provision of space to address emotional issues raised by coercion [14].

Only few studies have investigated the direct effect of post-coercion review sessions on subjective perception of coercion. The vast majority of these works is based upon qualitative data underlying the subjective benefits of such interventions and clarify the central role of emotional support aspects of post-coercion review [16,17].

Based on the theoretical background and the practical experiences made with a new recovery-oriented model of care, the “*Weddinger Modell*” [18], a guideline for a structured, post-coercion review session was developed by a multiprofessional working group. This guideline was evaluated in a pilot study showing that it was considered as a helpful tool and appraised by service users and staff members [19].

The present multicenter randomized-controlled trial aimed at evaluating the effects of standardized post-coercion review sessions on subjectively experienced coercion, also considering known influencing factors like gender and age. Participants were randomized to either receiving a standardized post-coercion review session or to standard care. It was hypothesized that the additional provision of the intervention would reduce the subjective experience of coercion throughout the hospital stay and regarding the index coercive intervention compared to standard care.

## Methods

### Design

The study was designed as a multicenter, two-armed, randomized controlled trial (ClinicalTrials.gov ID NCT03512925). The project was approved by the ethics committee of the Charité Universitätsmedizin Berlin (No. EA1/158/17). The authors assert that all procedures contributing to this work comply with the ethical standards of the relevant national and institutional committees on human experimentation and with the Helsinki Declaration of 1975, as revised in 2008.

### Participating clinics

All public psychiatric hospitals in Berlin were contacted through their head of departments. Six clinics providing acute psychiatric care for a defined catchment area agreed to take part in the present study.

### Participants

Participants were recruited on general psychiatric wards that routinely perform coercive measures. We included participants aged between 18 and 65, diagnosed with psychotic disorder (ICD-10: F1x.5, F2x, F30.2, and F31.2), who experienced at least one coercive measure (mechanical restraint, seclusion, and coerced medication on court order) during their hospital stay. People discharged within 24 h after admission, presenting severe cognitive deficits or limited knowledge in German were excluded from participation.

### Recruitment, randomization, and study procedure

Designated contact staff members on each ward were contacted by telephone daily to identify people who met inclusion criteria. Since the intervention only slightly differed from usual standards of care and since many potential participants were unable to consent to participation at the time of the first coercive measure, a randomization procedure as described by Zelen was used to avoid recruitment bias [20,21]. Following this method, potential participants meeting inclusion criteria were randomized after the first coercive measure to either the intervention or the control group. A block randomization with periods of eight on each ward was used, allocation status was concealed using sealed envelopes. The allocation was communicated to the ward’s contact person by telephone. Staff members, research team and participants were thus unblinded. For each randomized person, information about age, gender, type of coercive measure, and diagnosis were provided by the contact person to the research team. Potential participants were contacted and informed about the study by the research team in the course of their inpatient stay, when capacity to consent was restored. The assessment took place shortly before discharge, after receiving written informed consent.

Regarding the adherence to protocol, information regarding the reflecting review sessions that took place were communicated to the research team by the wards’ contact staff members. Daily contacts ensured the monitoring of the foreseen intervention and the planning of the study assessment. Additionally, we asked participants if they had received a post-coercion review session. Similarly, participants of the control group were asked whether some kind of post-coercion conversation had been initiated.

### Intervention: Standardized post-coercion review session

Participants allocated to the intervention group were offered with a standardized post-coercion review session conducted by trained staff members of the ward [19]. The session was repeatedly offered until discharge, as it was shown that the preferred moment to participate varies between individuals and should be freely determined by them. Although initially designed to be performed promptly after the first occurrence of coercion, results of our pilot study indicated that most patients were initially emotionally and clinically unable to participate in the interview. Information regarding the conducted post-coercion review is summarized in Table 1.

**Table 1.** Description of the post-coercion review session.

*Participants*: patient, staff member actively involved in the decision to use coercion, moderating staff member not directly involved in the coercive situation. Patients are encouraged to invite any person of trust or another member of staff or peer workers to participate. The moderator conducts the interview warranting the structure and completion of the interview, as well as inviting the patient to express his or her perception and feelings about the coercive measure and the situation that led to the coercive measure	
*Duration*: approximately 30–40 min	
*Procedure*:	
	1. Participants are asked to describe their perception of the crisis situation which lead to the eventual use of coercion and the coercive measure itself. Therefore, a process of sharing of patients’ and staff members’ perspectives is initiated
	2. The moderator asks open-ended questions to address following issues: alternatives to coercion, personal wishes during and after the coercive intervention, intelligibility of the reasons for the use of coercion. During this phase, the moderator facilitates the dialogue between all participants
	3. The conditions of an optimal pursuit of care are addressed
	4. At the end of the interview, the patient is offered the opportunity to include the conclusions of the interview in a joint crisis plan or an advance directive
The review session does not target an agreement on the necessity and justification of the coercive measure. It aims at giving all participants the opportunity to express their subjective experience, reflect on the past events and consider the different perspectives involved. Thus, the interview should contribute to reinforcing or repairing the therapeutic relationship and allow for an improved mutual understanding and respect	

Participating teams underwent a training session before study begin to ensure the correct application of the developed guideline. Training included information about the scientific background and the conduction of the intervention as well as role plays.

### Control intervention: Standard treatment

Participants allocated to the control group received usual treatment which sometimes comprised conversations about experienced coercive measures. However, none of these conversations in routine treatment followed determined standards.

### Measures

#### Sociodemographic and illness characteristics

Data regarding age, gender, socioeconomic status and migration status were collected during the assessment interview. Information about previous experiences of coercion and post-coercion reviews were collected as well.

#### Clinical data

Psychiatrists in charge of the participants completed the Global Assessment of Functioning scale (GAF) [22] and the Clinical Global Impression Severity scale (CGI-S) [23] for each participant regarding the time of the first coercive measure. To simplify symptoms assessment and reduce the amount of missing data, psychiatrists rated the severity of the following symptoms clusters on four-point Likert scales (absent, mild, moderate, and severe): positive symptoms, negative symptoms, global symptomatology, mania, depression, and lack of insight.

#### Objective use of coercion

Information about the type and number of coercive measures experienced by the participants during the index hospital stay was retrieved from the participants’ medical records.

#### Subjective coercion throughout the hospital stay

The global level of perceived coercion throughout the hospital stay was assessed using the German versions of the adapted MacArthur admission experience scale (AES) and the coercion ladder (CL).

The AES, originally designed to evaluate the level of perceived coercion linked to the admission process, was translated into German and adapted to analyze the perception of perceived coercion throughout the hospital stay. The AES comprises 23 items rated on a one- to five-point scale [24]. The first 15 items are allocated to three subscales: “perceived coercion” (five items), “negative pressures” (six items), and “process exclusion” (four items). The added scores of these three subscales form the AES-2 score. The last eight items build the subscales “treatment effectiveness” (four items) and “procedural justice” (four items) which are part of the AES-1 score. Higher AES-1 and AES-2 scores represent higher levels of perceived coercion or lower appraisal of received care, respectively [25].

The CL consists of a visual analogue scale ranging from 1 to 10, with higher values indicating higher levels of perceived coercion during hospital stay [26,27]. The CL was shown to parallel the results of the “perceived coercion” subscale (AES-PC) of the AES but seems to offer a more favorable administration and discrimination of higher levels of perceived coercion [4]. For the purpose of the present study, the introductory text of the CL was adapted in order to address the level of perceived coercion experienced during the whole inpatient stay.

#### Subjective coercion in relation to the experienced coercive intervention

The subjective perception of the burden occasioned by the specific coercive measure that was the subject of the post-coercion review was assessed using the coercion experience scale (CES) [28]. The CES is a self-rating instrument originally designed to compare the coerciveness of different coercive interventions. It features patients’ viewpoints on restriction of personal autonomy, human rights and the degree of suffering during the coercive intervention, in addition to numerous associated stressors on a five-point Likert scale. Psychometric studies of the CES have proven satisfying reliability and validity [28,29]. The sum score was utilized for analyses described below.

### Statistics

Using Zelen’s design, an intention-to-treat analysis based on the randomization results had to be restricted to those participants who consented to take part in the study. This main sample (“as consented”) was established and included participants regardless of study protocol violations. Sociodemographic and clinical characteristics were compared using Chi-square or Fisher’s exact test for categorical variables and Mann–Whitney-test for ordinal variables.

We conducted MANCOVA to analyze the main effects of the independent factors randomization status (post-coercion reflecting review session yes/no) and gender as well as their interaction on the main dependent variables AES 1 and AES 2. Age was integrated in the analysis as a covariate. Post hoc univariate analyses of variance (ANOVA) were performed using Bonferroni correction. Box’s test of equivalence of covariance matrices and Levene’s test of equality of variances were not statistically significant.

We conducted a similar ANCOVA to analyze the differences of the CL scores between the two study groups, using the randomization status, gender, as well as their interaction, as independent factors, and age as a covariate.

As to CES scores, ANCOVA was performed, using randomization status, gender, and the nature of the index coercive measure, as well as the interactions between randomization status and gender and between randomization status and the index coercive measure, as independent factors and age as covariate. The nature of the index coercive measure was integrated in order to account for the original purpose of the CES. As the number of forced medication incidents was comparatively very small, we chose to exclude those cases from analysis, leaving only seclusion and restraint as categories.

To account for protocol violations, we performed a sensitivity analysis based on a per-protocol sample, including all participants who had received the intervention (post-coercion review session) or the control condition as intended by randomization.

Statistical calculations were carried out using IBM SPPS Statistics 25. Statistical significance was defined at a two-sided *p* < 0.05.

## Results

### Sample description

**Figure 1.**
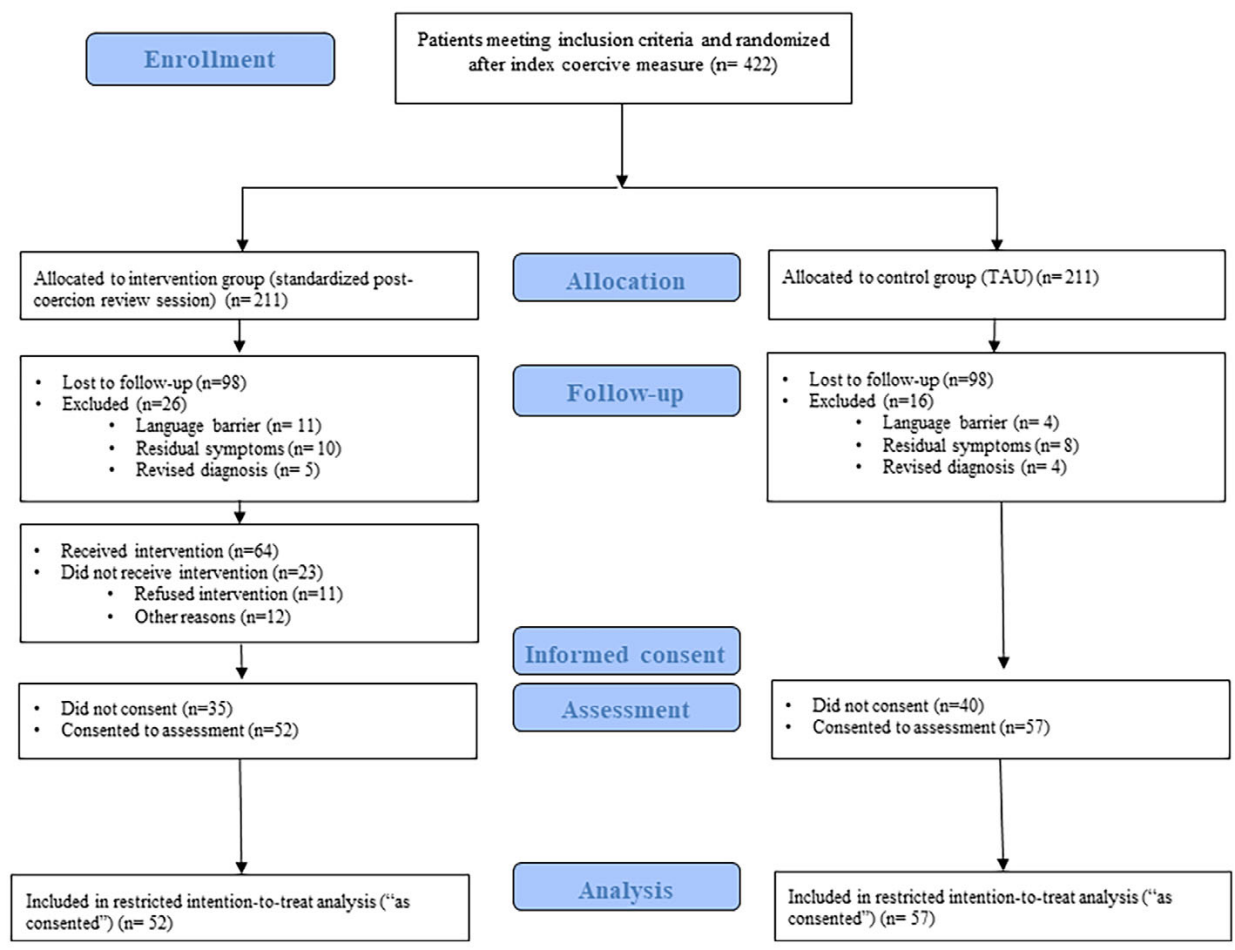
Study flowchart (adapted from the CONSORT diagram).

Overall, 422 participants were randomized after initial experience of a coercive measure (intervention group = 211; control group = 211). The randomization chart is shown in Figure 1. In both groups, 98 participants could not be solicited to participate because of early, unplanned discharge, absconding, or communication issues with the participating wards.

A total of 109 persons consented to participate (inclusion rate: 25.8%). Thus, 52 participants in the intervention group and 57 in the control group were included in the intention-to-treat analysis.

Forty-eight participants received a post-coercion reflecting review session according to clinical documentation; however, among them, eight participants reported having received no intervention. In the control group, 44 participants received no post-coercion review and 13 participants were offered nonstandardized post-coercion review. Accordingly, 92 participants were included in the sample used for the per-protocol analysis (intervention group: 48, control group: 44).

The sociodemographic and clinical characteristics of the samples that entered the restricted intention-to-treat (“as consented”) and the per-protocol analysis are summarized in Table 2. No significant group differences were found.

**Table 2.** Sociodemographic characteristics of the studied samples.

	Restr. intention-to-treat (“as consented”) (*n* = 109)		Per-protocol (*n* = 92)	
	Control	Intervention	Control	Intervention
	*n* = 57	*n* = 52	*n* = 44	*n* = 48
Age (yrs) *M (SD)*	39.11 (11.36)	38.54 (14.27)	38.66 (11.28)	39.02 (14.11)
Gender				
Female *n* (%)	26 (45.6%)	28 (53.8%)	20 (45.5%)	26 (54.2%)
Male *n* (%)	31 (54.4%)	24 (46.2%)	24 (54.5%)	22 (45.8%)
Hist. of migration *n* (%)	*n = 54*	*n = 49*	*n = 42*	*n = 45*
Yes *n* (%)	11 (20.4%)	17 (34.7%)	10 (23.8%)	14 (31.1%)
No *n* (%)	43 (79.6%)	32 (65.3%)	32 (76.2%)	31 (68.9%)
Incap. benefits *n* (%)	*n = 55*	*n = 44*	*n = 43*	*n = 40*
Yes	16 (29.1%)	12 (27.3%)	10 (23.3%)	11 (27.5%)
No	39 (70.9%)	32 (72.7%)	33 (76.7%)	29 (72.5%)
Level of education *n* (%)	*n = 55*	*n = 42*	*n = 43*	*n = 39*
No degree	4 (7.3%)	3 (7.1%)	3 (7.0%)	2 (5.1%)
Lower sec. education	9 (16.4%)	7 (16.7%)	6 (14.0%)	7 (17.9%)
Higher sec. education	15 (27.3%)	12 (28.6%)	14 (32.6%)	12 (30.8%)
High school graduation	11 (20.0%)	5 (11.9%)	9 (20.9%)	4 (10.3%)
Vocational college	7 (12.7%)	6 (14.3%)	4 (9.3%)	6 (15.4%)
University	9 (16.4%)	9 (21.4%)	7 (16.3%)	8 (20.5%)
Diagnosis *n* (%)				
F19.x5, F30.2, F31.2	10 (17.5%)	13 (25.0%)	7 (15.9%)	13 (27.1%)
F2.x	47 (82.5%)	39 (75.0%%)	37 (84.1%)	35 (72.9%)
Clinical parameters	*n = 53*	*n = 51*	*n = 41*	*n = 47*
GAF *M (SD)*	28.49 (12.42)	26.27 (13.28)	29.93 (12.73)	26.40 (13.67)
CGI-S *M (SD)*	5.53 (0.72)	5.80 (0.57)	5.49 (0.78)	5.79 (0.59)
*Symptom severity M (±SD)*				
Positive sympt.	2.43 (0.75)	2.27 (0.94)	2.34 (0.79)	2.28 (0.95)
Negative sympt.	1.21 (0.88)	1.18 (0.95)	1.17 (0.86)	1.17 (0.96)
Global sympt.	2.45 (0.64)	2.43 (0.70)	2.41 (0.67)	2.38 (0.71)
Mania	1.34 (1.13)	1.29 (1.24)	1.24 (1.14)	1.28 (1.25)
Depression	0.58 (0.86)	0.47 (0.67)	0.54 (0.78)	0.43 (0.65)
Lack of insight	2.30 (0.87)	2.29 (0.97)	2.17 (0.89)	2.28 (0.97)
Past coercion *n* (%)	*n = 56*	*n = 52*	*n = 43*	*n = 48*
Yes	37 (66.1%)	35 (67.3%)	28 (65.1%)	33 (68.8%)
No	19 (33.9%)	17 (32.7%)	15 (34.9%)	15 (31.3%)
Previous post-coercion review *n* (%)	*n* = 37	*n* = 36	*n* = 28	*n* = 34
Yes	3 (8.1%)	5 (13.9%)	0 (0.0%)	5 (14.7%)
No	34 (91.9%)	31 (86.1%)	28 (100.0%)	29 (85.3%)
Duration of index stay (days) *M (SD)*	54.69 (38.81)	70.10 (45.93)	52.95 (36.54)	69.56 (46.58)
Index coercive intervention *n* (%)				
Restraint	37 (64.9%)	31 (59.66%)	30 (68.2%)	27 (56.3%)
Seclusion	15 (26.3%)	18 (34.6%)	11 (25.0%)	18 (37.5%)
Forced med. on court order	5 (8.8%)	3 (5.8%)	3 (6.8%)	3 (66.3%)

Abbreviations: M, mean; SD, standard deviation.

### Time of intervention

Participants randomized to the intervention group received the foreseen review session at a median of 28.5 days after the initial coercive measure.

### Parameters of subjective experienced coercion

All results are summarized in Tables 3 and 4.

**Table 3.** Mean AES 1, AES 2, and CL.

	Restr. intention-to-treat (“as consented”)								Per-protocol							
	Control group *M* (SD)				Intervention group *M* (SD)				Control group *M* (SD)				Intervention group *M* (SD)			
	AES 1	AES 2	CL	CES	AES 1	AES 2	CL	CES	AES 1	AES 2	CL	CES	AES 1	AES 2	CL	CES
*m*	4.70 (2.04)	8.60 (2.97)	5.57 (3.06)	93.94 (28.65)	5.70 (2.19)	9.05 (3.14)	6.22 (3.19)	97.58 (31.79)	5.02 (2.06)	9.22 (2.92)	6.04 (3.18)	90.83 (28.48)	5.95 (2.11)	9.29 (3.19)	6.00 (3.43)	97.00 (31.94)
*f*	5.91 (2.68)	10.44 (3.14)	6.80 (3.45)	102.51 (36.67)	4.61 (2.48)	8.35 (3.77)	5.39 (3.27)	94.05 (30.41)	6.50 (2.68)	11.12 (2.84)	7.89 (3.02)	109.09 (36.06)	4.18 (2.22)	8.06 (3.53)	4.88 (3.19)	71.40 (28.97)
Total	5.27 (2.42)	9.47 (3.16)	6.13 (3.27)	97.22 (31.85)	5.11 (2.39)	8.67 (3.49)	5.76 (3.23)	95.61 (30.70)	5.72 (2.46)	10.12 (3.00)	6.88 (3.21)	97.93 (32.42)	4.88 (2.32)	8.53 (3.42)	5.33 (3.29)	93.95 (31.58)

*Note:* Scores across study groups in the different study samples displayed by gender.

*Abbreviations: AES, MacArthur admission experience survey; CES, coercion experience scale; CL, coercion ladder; M, mean; SD, standard deviation.*

**Table 4.** Descriptive statistics and results of the performed univariate ANCOVAs.

	Restr. intention-to-treat (“as consented”)				Per-protocol			
	df	*F*	*p*	Part. *η*^2^	df	*F*	*p*	Part. *η*^2^
*CL*								
Age	1	2.94	0.090	0.028	1	4.06	0.047*	0.050
Intervention	1	0.45	0.504	0.004	1	5.25	0.025*	0.064
Gender	1	0.16	0.688	0.002	1	0.43	0.516	0.005
Intervention × gender	1	2.95	0.089	0.028	1	4.21	0.044*	0.052
Error	101				77			
Total	106				82			
*AES 1*								
Age	1	2.38	0.126	0.024	1	1.78	0.186	0.024
Intervention	1	0.15	0.701	0.002	1	1.96	0.166	0.026
Gender	1	0.02	0.878	<0.001	1	0.05	0.832	0.001
Intervention × gender	1	6.63	0.012*	0.065	1	9.62	0.003**	0.116
Error	96				73			
Total	101				78			
*AES 2*								
Age	1	4.50	0.037*	0.045	1	6.69	0.012*	0.084
Intervention	1	1.89	0.172	0.019	1	5.16	0.026*	0.066
Gender	1	0.82	0.368	0.008	1	0.34	0.562	0.005
Intervention × gender	1	4.51	0.036*	0.045	1	4.91	0.030*	0.063
Error	96				73			
Total	101				78			
*CES*								
Age	1	1.04	0.310	0.012	1	6.03	0.017*	0.089
Intervention	1	0.92	0.340	0.011	1	2.14	0.148	0.033
Gender	1	0.62	0.434	0.007	1	2.81	0.099	0.043
Index coercive measure	1	6.17	0.015*	0.069	1	11.12	0.001*	0.152
Intervention × gender	1	0.68	0.412	0.008	1	1.58	0.214	0.025
Intervention × coerc. measure	1	1.925	0.169	0.023	1	1.89	0.178	0.029
Error	83				62			
Total	90				69			

Abbreviations: AES, MacArthur admission experience survey; CES, coercion experience scale; CL, coercion ladder; df, degrees of freedom; *F,* ANOVA *F*-value.

* *p* < 0.05;

** *p* < 0.01.

#### MacArthur admission experience survey

##### Restricted intention-to-treat analysis (“as consented”)

Using Pillai’s trace, a significant interaction effect between intervention and gender was identified, *V* = 0.067, *F*(2,95) = 3,416, *p* = 0.037, partial *η*^2^ = 0.067. There was no significant main effect of the post-coercion review session on the dependent variables AES-1 and AES-2, *V* = 0.025, *F*(2,95) = 1,201, *p* = 0.305, partial *η*^2^ = 0.025. Similarly, no main effects of the independent variable gender or the covariate age were found.

Post hoc ANOVAs revealed a significant interaction effect between intervention and gender for both the AES-1 and AES-2. Simple effects analyses revealed that the intervention significantly reduced the perception of coercion in women (AES-1: *F*(1,96) = 4,447, *p* = 0.038, partial *η*^2^ = 0.044; AES-2: *F*(1,96) = 6,202, *p* = 0.014, partial *η*^2^ = 0.061) but not in men (AES-1: *F*(1,96) = 2,370, *p* = 0.127, partial *η*^2^ = 0.024; AES-2: *F*(1,96) = 0.278, *p* = 0.599, partial *η*^2^ = 0.003). No significant main effect of the intervention or gender was found. A significant main effect of the covariate age regarding AES-2 scores was found. Older age was associated with lower AES-2 scores.

##### Sensitivity analysis

As to the per-protocol analysis, multivariate analysis yielded a significant interaction effect between the intervention and gender, *V* = 0.117, *F*(2,72) = 4,779, *p* = 0.011, partial *η*^2^ = 0.117. No significant main effect of the intervention or gender was evident when comparing both groups. However, a significant main effect of age (*V* = 0.089, *F*(2,72) = 3,402, *p* = 0.039, partial *η*^2^ = 0.086) was identified.

At the univariate level, post hoc analysis showed a significant interaction effect between intervention and gender for both AES-1 and AES-2. Once again, simple effects analyses showed a significant influence of the intervention on both AES subscales in women (AES-1: *F*(1,73) = 11,100, *p* = 0.001, partial *η*^2^ = 0.132; AES-2: *F*(1,73) = 11,020, *p* = 0.001, partial *η*^2^ = 0.131) but not in men (AES-1: *F*(1,73) = 1,328, *p* = 0.253, partial *η*^2^ = 0.018; AES-2: *F*(1,73) = 0.002, *p* = 0.969, partial *η*^2^ < 0.001). As to other univariate analyses, results showed a reduction of the level of perceived coercion according to the AES 2 scores among participants, who received the foreseen standardized post-coercion review session compared to controls. No effect of gender was found.

Similarly to the analysis of the “as consented” sample, a significant main effect of the covariate age on AES-2 scores was found, whereby decreased AES-2 scores were seen in older participants.

#### Coercion ladder

##### Restricted intention-to-treat analysis (“as consented”)

The performed two-way ANCOVA showed no significant effect of the standardized post-coercion review session. The main effects of gender, age and the interaction effect of post-coercion review and gender did not reach the significance threshold.

##### Sensitivity analysis

The per-protocol analysis showed a significant main effect of the foreseen intervention on the mean CL score. A significant interaction effect between intervention and gender (*F*(1.77) = 4,210, *p* = 0.044, partial *η*^2^ = 0.052) was confirmed. The foreseen intervention had a significant effect regarding female (*F*(1.77) = 10,031, *p* = 0.002, partial *η*^2^ = 0.115), but not male participants (*F*(1.77) = 0.027, *p* = 0.869, partial *η*^2^ < 0.001). No significant main effect of gender was found.

The covariate age was significantly related to the CL scores, with the level of subjective coercion decreasing with older age.

#### Coercion experience scale

##### Restricted intention-to-treat analysis (“as consented”)

Participants in the intervention group showed slightly lower CES mean scores (M = 95.61, SD = 30.70) compared to those in the control group (M = 97.22, SD = 31.85). Participants who experienced restraint (control: M = 98.35, SD = 33.01; intervention: M = 105.45, SD = 24.79) showed higher CES scores compared to those who experienced seclusion (control: M = 94.81, SD = 30.19; intervention: M = 75.23, SD = 32.53).

The two-way ANOVA yielded no significant main effect of post-coercion review (*F*(1,83) = 0.920, *p* = 0.340) or gender (*F*(1,83) = 0.620, *p* = 0.434). There was a significant main effect of the nature of the index coercive measure (*F*(1,83) = 6.170, *p* = 0.015). There was neither a significant interaction effect between post-coercion review and gender, nor between post-coercion review and kind of the coercive measure.

##### Sensitivity analysis

In the per-protocol analysis, no significant main effect of post-coercion review (*F*(1,62) = 2.144, *p* = 0.148) or gender (*F*(1,62) = 2.807, *p* = 0.099) could be shown. There was again a significant main effect of the kind of experienced coercive measure (*F*(1,62) = 11.120, *p* = 0.001). No interaction effect between intervention and gender or between intervention and the kind of coercive measure was found.

## Discussion

The results of this randomized controlled trial could not show a significant main effect of post-coercion review sessions on the experience of subjective coercion during an inpatient stay. Statistical analyses within the sample of all randomized participants who had consented to the study examination failed to yield a significant effect of the intervention on AES and CL scores. Similarly, no effect of the intervention regarding CES scores was found. It therefore has to be questioned whether a single intervention can be deemed sufficient to process a potentially traumatic event like a psychiatric coercive intervention. Results of the pilot study indicate a positive appraisal of the intervention by patients, but also show that there is heterogeneity regarding its timing, content and felt necessity[19]. Moreover, subjective coercion was mainly evaluated with respect to the whole length of the hospital stay, and therefore a whole spectrum of other influential factors like staff attitudes, treatment milieu and concepts, kind and intensity of other therapeutic interventions must be considered. Future research should include a broader range of predictors to capture the determinants of perceived coercion in psychiatry.

However, further analysis revealed interesting results, showing a significant interaction between intervention and gender regarding subjective perceptions of coercion represented by AES 1 and AES 2 scores. Sensitivity analyses confirmed this result in the per-protocol sample and yielded positive main and interaction effects regarding perceived coercion as measured by the CL. These results add to the conclusions of previous works which underlined the positive perception of post-coercion reviews reported by patients [16,30]. The opportunity to reflect on an escalating interpersonal situation together with staff members directly involved in the situation might be linked to its positive effect. Reductions of AES scores found in this study suggest that review sessions may help to reduce experienced negative feelings and can change the perception of the treatment fairness. The setting of the session may enable service users and staff members to acknowledge the gravity of feelings usually experienced during coercive measures. Moreover, review sessions can facilitate the repair and reinforcement of the therapeutic relationship. This is partly suggested by our results regarding the increasing perception of procedural justice and fairness as an effect of the intervention. Besides, the present RCT has also shown a significant reduction of symptoms of PTSD [31]. There again, the mutual reflection process, including the discussion of the motives for the use of coercion, initiated by the review session seemed to mitigate the risk of developing post-traumatic symptoms.

As expected, younger age was associated with higher levels of perceived coercion. This might indicate that younger patients who arenot used to psychiatric settings are more prone to experience inpatient care as harmful or coercive than older patients, who might have experienced even more coercive treatments and settings in the past.

The performed analyses showed that post-coercion review sessions were significantly associated with lower levels of subjectively perceived coercion and the experience of greater fairness and justice in female participants. A previous study yielded that male service users are more prone to experience restraint as compared to women[32], and accordingly, mechanical restraint was more frequently applied in male rather than in female participants in our sample. Despite this fact, female participants in the control group, but not in the intervention group, exhibited higher levels of subjective coercion compared to males at the end of their treatment. Higher levels of perceived coercion among women have already been reported elsewhere [9,10] but to our knowledge, our study is the first to describe gender-specific effects of a therapeutic intervention in this domain.

As a possible explanation, it could be speculated that men more often than women may have experienced coercion or even exerted violence during their treatment, but also in their living or social environment. For this reason, they might probably experience coercion as less offending and as a proportionate response to their violent behavior. The perception of coercion as inevitable might thus explain the poorer effect of review sessions in men, and their lower levels of perceived coercion. Additionally, alcohol or drug use in the context of an escalating situation seems to be more common in men suffering from psychotic disorders [33]. This might foster the perception of a violent situation as less coercive, or even cause amnesia. An alteration of focus and efficacy of the review sessions thus seems plausible in this context.

A greater subjective perception of coercion in female samples might also be related to partly socially influenced behaviors like a more profound emotional responsiveness toward violence or the greater tendency to acknowledge negative feelings and judgments about treatment [34]. Women might also show a greater willingness to emotionally engage in a post-coercion review session than men, and their benefit from it might be linked to a greater degree of psychological mindedness [35]. Eventually, a greater acknowledgment of the therapeutic aspects of the review sessions might be impacted by more pronounced socially desirable response tendencies in females. Women are also more frequently subject to sexual offenses and violence, which all bear a serious traumatic potential that can be reactivated within the psychiatric setting and thus impact their perception of coercion.

These findings suggest the need to differentiate methods of addressing the experience of men and women on psychiatric wards. Further research is needed to assess potential gender differences regarding formal, informal, and subjectively experienced coercion.

It is noteworthy that the significant effect of standardized post-coercion review session on the burden of symptoms of post-traumatic stress was not significantly influenced by gender [31]. This could indicate that although the consequences of coercion and its subjective perception are played down by men because of socially influenced behaviors and thought patterns, the impact of coercive measures on the neuro-vegetative level does not differ between men and women.

As to the level of coercion experienced in direct relation to the applied coercive measures, our analysis showed that restraint was associated with higher levels of subjective coercion compared to seclusion. Although a first RCT did not show any differences between restraint and seclusion, a follow-up study by Steinert et al. also showed higher CES scores among patients who experienced restraint compared to seclusion [29,36]. The present work thus confirms the high coercive potential of mechanical restraint. As to the effect of post-coercion review, the lack of effect of the intervention should not be considered as surprising, as it could be hypothesized that such an intervention does not have the power to retrospectively influence the factual circumstances and the respective burden experienced during a coercive measure, which constitute the main focus of the CES.

Some limitations of the present work must be considered. Firstly, the randomization procedure was chosen to fit the studied population of severely ill people experiencing coercive measures on psychiatric wards and to allow their recruitment. The targeted study population is per definitionem unable to consent, and therefore Zelen’s design had to be applied. As the focused outcome parameters of subjective coercion exceed measures that are collected within clinical routine, the main sample for the analysis consisted of patients, who had been randomized and also actively consented to the study, thus limiting a full intention-to-treat approach. This is important, as only about 25% of patients who had experienced a coercive intervention and were consecutively randomized could be included in the analysis. In addition to the denial of consent, difficulties in contacting potential participants, either because of persistent symptomatology, early, unexpected discharge against medical advice, or communication issues hampered effective recruitment. In many cases coercive interventions were linked to emergency situations before or during admission, and for instance in cases of concomitant substance abuse the reasons to be involuntarily committed to a psychiatric hospital were no longer present the following days. However, this problem reflects on the one hand the daily reality of acute psychiatric wards and the uttermost difficulty to conduct a RCT within this setting; on the other hand, it illustrates the implementation difficulties of a clinical intervention for severely ill patients in the context of acute care. Moreover, is must be noted that post-coercion review sessions are legally required at least in some German federal states, and efforts must be made to guarantee the provision of this intervention also following inpatient hospital treatment. Flexible settings including home treatment and a maximum of therapeutic continuity may facilitate the implementation of the intervention. Alongside the limitations of the statistical power of the analysis, recruitment impediments may have led to selection bias. It is probable that study participants might have been more likely to have a minimally positive attitude toward psychiatry or ward staff, while patients who rejected the offer of hospital support and left the ward as early as possible might have experienced an even higher extent of subjective coercion.

Secondly, and as mentioned, some participants of the intervention group did not receive post-coercion review (or a nonconform version), and some individuals of the control group received an active post-coercion intervention from staff. Most interestingly, eight participants stated that they had not received a post-coercion review, although staff members witnessed it. This could be explained by relational difficulties, florid delusional symptomatology or probably, by choosing a point of time for the intervention, when the person could not fully engage in the process.

Thirdly, the intervention took place after a relatively long period of time after the initial coercive measure, most probably due to the emotional and clinical readiness required to undergo an intervention of this kind. This underlines the necessity to address the issues service users face after a coercive measure and to develop other formats of post-coercion review, specifically tailored to acknowledge service users’ individual needs and therapy phase in this context.

Finally, it is worth mentioning that the used instruments AES and CL were not originally designed to evaluate the perception of coercion throughout the hospital stay. Although the used adaptations yielded interesting results, specific instruments are lacking that could capture the whole scope of experienced coercion in inpatient settings.

In conclusion, although the present study did not show a direct impact of post-coercion review sessions on subjective coercion, it is the first to indicate gender-related aspects of such an intervention. The results show that such an intervention can help to alleviate the negative experiences made in the context of psychiatric inpatient care and hopefully prevent their negative impact on the course of illness and treatment, especially among women. Results also indicate a relation between gender-specific aspects and the subjective experience of coercion. This needs to be addressed specifically in the future development and implementation of interventions aiming to reduce coercion.

## Data Availability

The data that support the findings of this study are available from the corresponding author, A.W., upon reasonable request.
